# A review of the Oriental genus *Eudeferunda* Chen, Yang & Wilson (Hemiptera, Fulgoromorpha, Achilidae) with description of one new species from Hainan, China

**DOI:** 10.3897/zookeys.313.5106

**Published:** 2013-06-27

**Authors:** Jian-Kun Long, Lin Yang, Xiang-Sheng Chen

**Affiliations:** 1 Institute of Entomology, Guizhou University, Guiyang, Guizhou, 550025, P.R. China; 2 The Provincial Key Laboratory for Agricultural Pest Management of Mountainous Region, Guizhou University, Guiyang, Guizhou, 550025, P.R. China

**Keywords:** Achilid, Fulgoroidea, Oriental region, planthopper, taxonomy

## Abstract

A new species of *Eudeferunda* Chen, Yang & Wilson, 1989 (Hemiptera: Fulgoromorpha: Achilidae: Plectoderini), *Eudeferunda alatea* Long & Chen **sp. n.** from Hainan, China, is described and illustrated. The generic characters are modified, including the addition of male genitalia characters. A key to the species of the genus is provided.

## Introduction

The achilid genus *Eudeferunda* (Hemiptera: Fulgoromorpha: Achilidae: Plectoderini) was established by Chen et al. (1989), and with *Eudeferunda lenita* Chen, Yang & Wilson, 1989 as its type species. To date, this genus contains only the type species. Here, we described and illustrated a new species: *Eudeferunda alatea* Long & Chen sp. n. from South China. A key to separate these two species of *Eudeferunda* is provided.

## Materials and methods

Specimens were collected by sweeping. Dry specimens were used for the description and illustration. External morphology was observed under a stereoscopic microscope and characters were measured with an ocular micrometer. The genital segments of the examined specimens were macerated in 10% KOH and drawn from preparations in glycerin jelly using a Leica MZ 12.5 stereomicroscope. Illustrations were scanned with Canon CanoScan LiDE 200 and imported into Adobe Photoshop CS3 for labeling and plate composition. Terminology for morphology following by Szwedo and Żyła (2009) and Bourgoin and Huang (1990). The type material of specimens are deposited in the Institute of Entomology, Guizhou University, Guiyang, Guizhou Province, China (IEGU).

## Taxonomy

### Eudeferunda

Chen,Yang & Wilson, 1989

http://species-id.net/wiki/Eudeferunda

Figs 1 – 28

Eudeferunda Chen et al. 1989: 16.

#### Type species.

*Eudeferunda lenita* Chen, Yang & Wilson, 1989, by original designation.

#### Diagnosis.

Small size. Head with eyes narrower than pronotum (0.7–0.8:1) (Figs 7, 19). Vertex produced before eyes about 0.4 times of its length, longer in middle line than wide at base (1.4:1), disk distinctly depressed, median carina basal half prominent, anterior half obsolete, anterior margin acutely rounded convex, lateral margins carinate, straight, diverging posteriorly, posterior margin in middle angulately convex (Figs 7, 19). Frons longer in middle line than widest part (1.25–1.4:1), basal margin subtruncate or truncate, about quarter length of widest part, lateral margins strongly foliate basally, slightly convex laterally, thence incurved into suture (Figs 8, 20). Postclypeus shorter than frons in middle line (0.4–0.6:1) (Figs 8, 20). Eyes incised beneath, not or slightly overlapping pronotum (Figs 7, 9, 19, 21). Ocelli detached from eyes. Antennae with pedicel subglobose. Pronotum moderately short, as long behind eyes as in middle line, disc with median carina shorter than lateral carinae (0.5:1) (Figs 7, 19). Mesonotum tricarinate, longer than vertex and pronotum together, lateral carinae slightly diverging posteriorly, middle carina apically obsolete (Figs 7, 19). Forewing 2.5–2.9 times as long as broad, CuA_1_ not convex strongly, Sc+R forking level of CuA_1_ fork, both slightly distally union of PCu with A_1_, clavus terminating at midway of forewing (Figs 10, 22). Post-tibia with a single lateral spine at basal 2/5, metatibio-tarsal formula of hind leg 8–7–6 (Figs 5–6). Anal segment relatively short, in dorsal view apical margin concave in middle (Figs 12, 23). Pygofer in lateral view distinctly longer ventrally than dorsally, anterior margin concave, posterior margin convex and produced a curved process directing downward (Figs 13, 24); in ventral view medioventral processes paired, median cleft deep (Figs 14, 25). Dorsal margin of gonostyle with a large triangular process laterad, thence laterad convex near base (Figs 15–16, 26). Male genitalia with phallobase submembraneous, tube-like, phallobase with dorsal and ventral lobes; ventral lobe of phallobase in ventral view cleft at apical margin in middle, each lateral side with a pointed processes, bilateral areas with thorns; in dorsal view, dorsal lobe of phallobase with apical margin broadly incised in middle; phallic appendages longer than phallobase about 3:1, gradually narrowing apically (Figs 17–18, 27–28).

#### Distribution.

Oriental region.

#### Key to species of *Eudeferunda* Chen, Yang & Wilson (male)

**Table:** 

1	Medioventral processes of pygofer digitate, gradually narrowing apically (Fig. 25); anal segment in dorsal view with base margin truncate and blunt laterally (Fig. 23); ventral lobe of phallobase with a couple of lateral processes near apex (Figs 27–28)	*Eudeferunda lenita*
–	Medioventral processes of pygofer with apex distinctly attenuate and bent towards outboard (Fig. 14); anal segment in dorsal view with basal margin slightly sinuate and angular laterally (Fig. 12); ventral lobe of phallobase with a couple of lateral processes at base 1/3 (Fig. 17)	*Eudeferunda alatea* sp. n.

### Eudeferunda alatea

Long & Chen sp. n.

urn:lsid:zoobank.org:act:7BB7C6C5-3FAA-4B0B-BCC2-AF560530F217

http://species-id.net/wiki/Eudeferunda_alatea

Figs 1 –18

#### Type material.

Holotype: 1♂, **China:** Hainan, Ledong, Jianfengling National Natural Reserve (18°41'N, 108°36'E), 16 Jan. 2011, J.-K Long. Paratypes: 1♂, 3♀♀,Hainan, Ledong, Jianfengling National Natural Reserve (18°41'N, 108°36'E), 13–16 Jan. 2011, J.-K Long; 1♀, Hainan, Ledong, Jianfengling National Natural Reserve (18°41'N, 108°36'E), 14 Jan. 2011, W.-B. Zheng (IEGU).

#### Etymology.

The species name is derived from the Latin word “*alate*”, indicating the phallobase with a pointedly alate process at base 1/3 of each lateral side.

#### Description.

Body length (from apex of vertex to tip of forewings): male 3.75– 3.80 mm (N=2), female 4.50–4.75 mm (N=4); forewing length: male 2.95–3.05 mm (N=2), female 3.55–3.75 mm (N=4).

**Figures 1–6.**
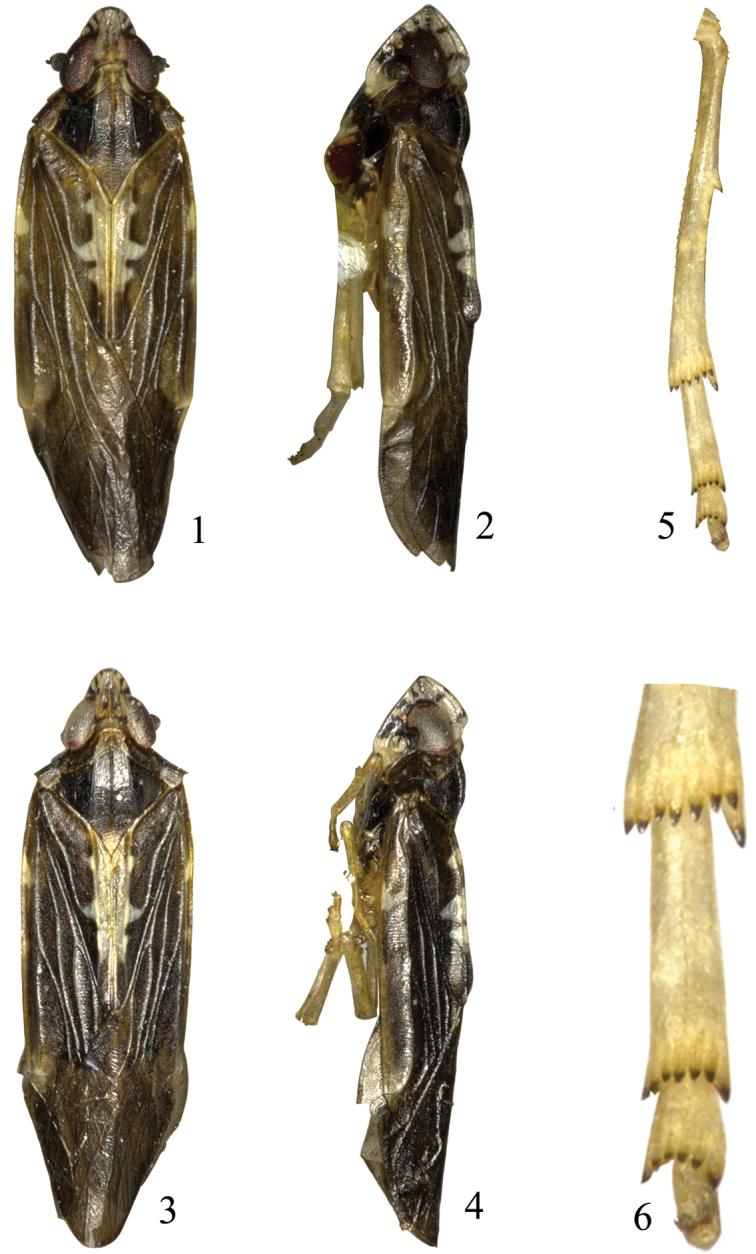
*Eudeferunda alatea* Long & Chen sp. n. **1** Male habitus, dorsal view **2** Male habitus, lateral view **3** Female habitus, dorsal view **4** Female habitus, lateral view **5** Hind tibia and tarsus **6** Apex of hind leg.

**Figures 7–18.**
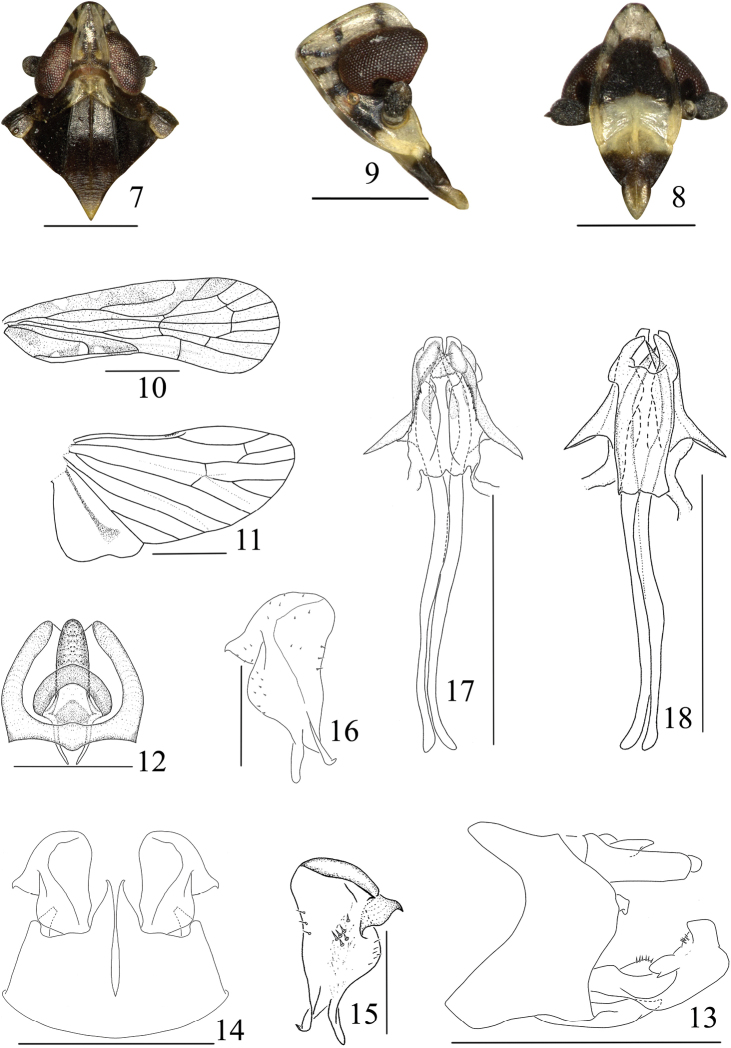
*Eudeferunda alatea* Long & Chen sp. n. **7** Head and thorax, dorsal view **8** Frons and clypeus **9** Head, lateral view **10** Forewing **11** Hindwing **12** Anal segment, dorsal view **13** Male genitalia, lateral view **14** Male genitalia, ventral view **15** Left gonostyle, dorsal view **16** Left gonostyle, ventral view **17** Aedeagus, ventral view **18** Aedeagus, dorsal view. Scale bars: = 1 mm (Figs **10–11**); 0.5 mm (Figs **7–9, 13–14, 17–18**); 0.2mm (Figs **12, 15–16**).

**Coloration.** Ivory white to black brown (Figs 1–9). Vertex ivory white, with black brown at base, longitudinal black brown stripe, gradually narrowing forward (except middle carina light brown) in middle, anterior and lateral margins brown, lateral margin with two transverse black brown stripes respectively at the level of anterior margin of eyes and base (Fig. 7). Frons with disk in middle black brown, basal and apical fourth ivory white, lateral margins with three big and one small fuscous spots (Fig. 8). Postclypeus with basal half ivory-white and apical half fuscous; preclypeus light brown except the base fuscous (Fig. 8). Genae ivory white, with three fuscous transverse stripes before eyes, two dorsad, area between ocellus and antenna fuscous, genae coloration at level of clypeus same the face (Fig. 9). Eyes reddish brown, ocelli pale reddish-brown (Fig. 9). Antennae fuscous (Figs 7–9). Pronotum dark brown, area between out side of two lateral carinae ivory white; mesonotum dark brown, with yellowish brown at apical angle (Fig. 7). Tegula dark brown, with dorsally lateral margin yellowish brown (Fig. 7). Forewing yellowish brown to dark yellowish brown, costal margin with three ivory white spots, clavus with three ivory white spots inside A_2_ and one longitudinal ivory white stripe between A_2_ and posterior margin (Fig. 10). Hindwing pale brown, veins pale brown. Legs pale brown (Figs 2, 4, 5–6). Abdomen dark brown.

**Head and thorax.** Head with eyes narrower than pronotum (0.81:1) (Fig. 7). Vertex long, broad across base than anterior (5.00:1), produced before eyes about 0.36 times of its length, width of vertex measured at base of middle line 0.66 times length along middle, longer in middle line than wide at base (1.41:1). Frons longer in middle line than widest part (1.25:1), basal margin subtruncate, middle carina gradually weakening basally (Fig. 8). Postclypeus shorter than frons in middle line (0.42:1) (Fig. 8). Rostum with apical segment longer than subapical one (1.47:1). Pronotum shorter than vertex in middle line (0.31:1). Mesonotum longer than pronotum in middle line (6.8:1), than vertex and pronotum together (1.61:1) (Fig. 7). Disc of tegula with several small longitudinal ridges (Fig. 7). Forewing 2.93 times as long as broad (Fig. 10). Hindwing 1.78 times as long as broad (Fig. 11).

**Male genitalia.** Anal segment in dorsal view slightly shorter than broad (0.90:1), basal margin slightly sinuate and angular laterally, apical margin concave (Fig. 12). Medioventral processes of pygofer paired, with apex distinctly attenuate and bent towards outboard, median cleft deep (Figs 13–14). Dorsal margin of gonostyle with a small dentiform process dorsad and a large triangular process laterad, thence laterad convex near base, apical margin broadly convex (Figs 15–16). Male genitalia with phallobase in ventral view (Fig. 17), ventral lobe cleft at apical margin in middle, bilateral areas hump-shaped and protuberated subapically, a pointedly alate process at base 1/3 of each lateral side, with its base of inside margin denticulated and apically extended; in dorsal view (Fig. 18), dorsal lobe with apical margin broadly incised in middle, bilateral margin ox-horn like produced. Phallic appendages longer than phallobase (3:1), gradually narrowing apically (Figs 17–18).

#### Distribution.

South China (Hainan).

#### Remarks.

This new species differs from *Eudeferunda lenita* Chen, Yang & Wilson, 1989 by: mesonotum dark brown, only with yellowish brown at apical angle (between lateral carinae white in *lenita*); forewing with three ivory white spots along costal margin (ivory white area among costal cell, Sc+R, and stigma in *lenita*); disk of tegula with several small longitudinal ridges (without in *lenita*); male pygofer of medioventral processes with apex distinctly attenuate and bent towards outboard (digitate and gradually narrowing apically in *lenita*); anal segment in dorsal view with basal margin slightly sinuate and angular laterally (base margin truncate and blunt laterally in *lenita*); ventral lobe of phallobase with a couple of lateral processes at base 1/3 (near apex in *lenita*).

### Eudeferunda lenita

Chen, Yang & Wilson, 1989

http://species-id.net/wiki/Eudeferunda_lenita

Figs 19–28

Eudeferunda lenita Chen et al. 1989: 16.

#### Distribution.

South China: Henchun (22°00'N, 120°44'E), Pingtung City, Taiwan.

#### Material examined.

No specimen has been collected by the authors.

**Figures 19–28.**
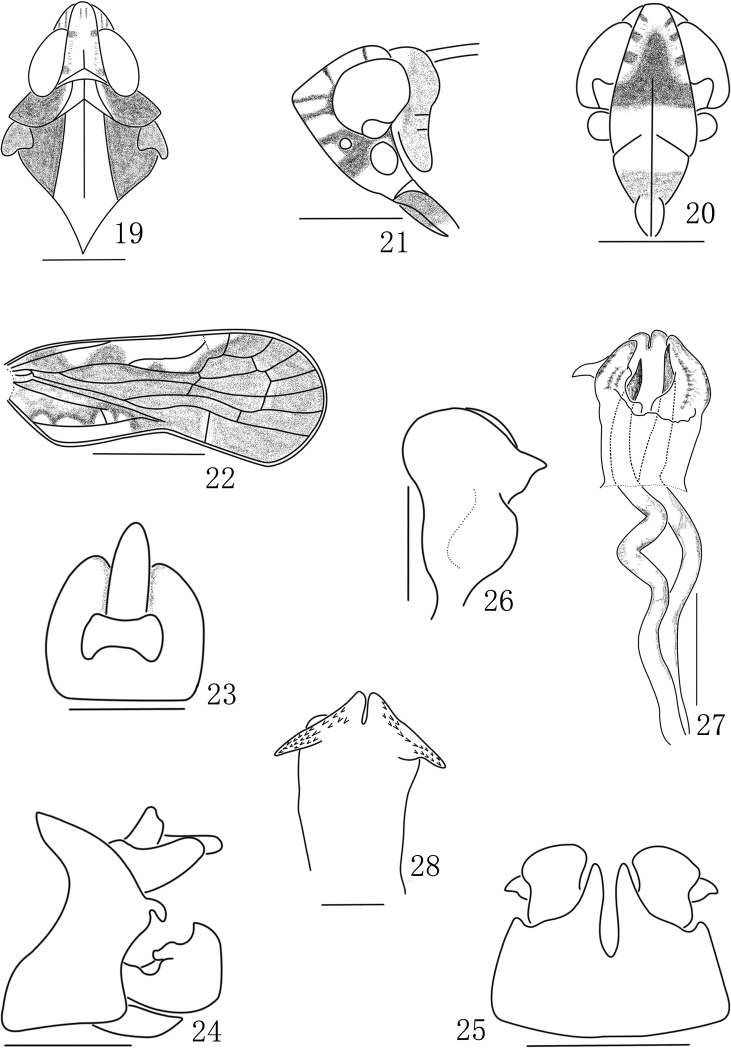
*Eudeferunda lenita* Chen, Yang & Wilson, 1989. **19** Head and thorax, dorsal view **20** Frons and clypeus **21** Head, lateral view **22** Forewing **23** Anal segment, dorsal view **24** Male genitalia, lateral view **25** Male genitalia, ventral view **26** Left gonostyle **27** Aedeagus, dorsal view **28** Phallobase, ventral view. Scale bars: = 1 mm (Figs **10–11**); 0.5 mm (Figs **7–9, 13–14, 17–18**); 0.2mm (Figs **12, 15–16**) (all after Chen et al. 1989).

## Supplementary Material

XML Treatment for Eudeferunda

XML Treatment for Eudeferunda alatea

XML Treatment for Eudeferunda lenita
